# Under forty hours of laboratory training may be sufficient for teaching basic microsurgical skills to novices

**DOI:** 10.1007/s00701-025-06704-3

**Published:** 2025-11-05

**Authors:** Adam Yousfi, Ville Vasankari, Anni Pohjola, Anna Maria Auricchio, Francesco Calvanese, Ahmad Hafez, Martin Lehecka

**Affiliations:** 1 Department of Neurosurgery, Helsinki University Hospital, University of Helsinki, Helsinki, Finland; 2 Papa Giovanni XXIII Hospital, Departement of Neurosurgery, Bergamo, Italy; 3 Azienda Ospedaliera Universitaria Pisana, Pisa, Italy

**Keywords:** Surgical training, Novices, Residents, Residency, Microsurgery, Neurosurgery, Exoscope

## Abstract

**Background:**

Structured laboratory-based microsurgical training is considered beneficial for junior residents before they begin to work in a neurosurgical operating room. The optimal duration of such training remains unclear. We studied the effect of a 40-h microsurgical laboratory course on the development of basic microsurgical skills among novices.

**Methods:**

A total of 27 medical students participated in the study. Seven students (5 men, 2 women) underwent a structured microsurgical laboratory training program over four weeks. 20 students (10 women, 10 men) served as an untrained control group. None of the students had prior experience in microsuturing or working under magnification. The intervention group practiced for two hours per day, five days per week, over a four-week period. Skill development was assessed at baseline, after two weeks, and after four weeks of training with a microsuturing test task, and was monitored continuously throughout the training period with a microscraping test task. Microsurgical performance was compared between the intervention and the control groups using a test task performed under a surgical exoscope after two weeks of training. This task assessed complex depth perception and hand–eye coordination through the precise placement of a microneedle into small, concealed targets.

**Results:**

For the microsuturing task the median time improved throughout the training (baseline: 44 min (interquartile range IQR = 22), 20 h training: 21 min (IQR = 6), 40 h training: 14 min (IQR = 4)). Similarly, the duration of the scraping task improved (baseline: 40 min (IQR 2), 20 h training: 15 min (IQR = 7), 40 h training: 13 min (IQR = 7)). The quality of work, including scraping accuracy, suture tightness and spacing, remained consistent in both tasks. In the exoscope task, the intervention group outperformed the control group (median task duration 6 min 54 s, IQR = 3 min vs 9 min 24 s, IQR = 6 min; *p* = 0.04).

**Conclusions:**

Novices demonstrated rapid improvement during a 40-h microsurgical laboratory training course. Even less than 40 h of structured practice might be enough for surgical novices, such as neurosurgery residents, to learn the most basic microsurgical skills prior to assisting in the operating room.

## Introduction

Modern microneurosurgery relies not only on magnification but also on operating techniques that protect the surrounding tissues and preserve normal anatomy [13]. Traditionally, microsurgical training has been based on a master-apprentice model, where trainees first observe, then gradually assist, and later perform similar surgeries themselves. Lately, the training is increasingly challenged by working hours regulations, limited number of operative cases and patient safety considerations. While theoretical knowledge is important, the early development of fine motor control and hand–eye coordination is crucial for surgical performance.

Microsurgical laboratory training has proven effective in improving the manual skills required in neurosurgery [1, 11, 15, 21, 22]. Acquiring basic microsurgical skills is often described as highly time-consuming, with some protocols recommending up to 7–8 h of daily training for several weeks [18]. In resource-limited settings, such an intensive schedule may be difficult to organize [7]. It remains unclear how much microsurgical laboratory training is sufficient before residents are adequately prepared to assist in the microsurgical operating theatre.

In this study, we aim to evaluate the effectiveness of a structured 40-h microsurgical laboratory training program for novices. First, we hypothesize that the trainees who receive laboratory training will demonstrate significant improvements in basic microsurgical performance and surpass their non-trained controls during 40 h of training. Second, we hypothesize that skill acquisition will plateau toward the end of the 40-h training period, indicating a potential threshold for early competence.

## Materials and methods

### Participants

We recruited 27 students drawn exclusively from the first and second years of medical school. The students had no prior experience in microsurgery, and none had previously used an exoscope or an operating microscope. They were divided into two groups, an intervention group (*n* = 7; 5 men, 2 women) and a control group (*n* = 20; 10 men, 10 women). The intervention group received 40 h of structured microsurgical training during one month period. The control group received no training. All participants reported normal 3D depth perception.

All participants gave their informed consent to participation. No information allowing identification of the participants was collected. Based on Finnish Legislation, pseudonymized datasets do not require participant consent.

### Study design

The intervention group (*n* = 7) was trained by six neurosurgeons (VV, AP, AH, AMA, FC and ML) and participated in daily training for two hours over four weeks. For each participant, we designed a guided 40-h individual training program which included the following modules: (A) microsuturing under a microscope (M320, Leica Microsystems®, Wetzlar, Germany), (B) microscraping under a microscope, and (C) manual dexterity test task with an exoscope (Aesculap AEOS®; BBraun, Melsungen, Germany), performed halfway through training (after 20 h). Exoscopes are novel magnifying devices in neurosurgery, which are considered noninferior to microscopes [2, 4]. The exoscope used in our study was equipped with a foot pedal.

The control group (*n* = 20) did not undergo this kind of structured training; they only performed the manual dexterity test task with an exoscope (C). The exoscope test task was preceded in both groups by a brief, on-location tutorial on exoscope use, and a few minutes of free-training with the exoscope, the test model and the necessary microinstruments.

#### Training of intervention group

The intervention group (*n* = 7) participated in 40-h microsuturing training over four weeks. First, they practiced on latex gloves using 6–0 and 7–0 thread (approximately 1.5 h/day). As suturing advanced, the students additionally practiced on suturing pads and 3D-printed models, and chicken wing anastomosis models. Chicken wing microanastomosis practice was conducted with 10–0 thread. All the aforementioned microsuturing tasks were considered free training and were not graded. Microsuturing development was tracked using a specifically designed 8-stitch test task, completed thrice during training.

##### Microsuturing test task (intervention group)

**Fig. 1:**
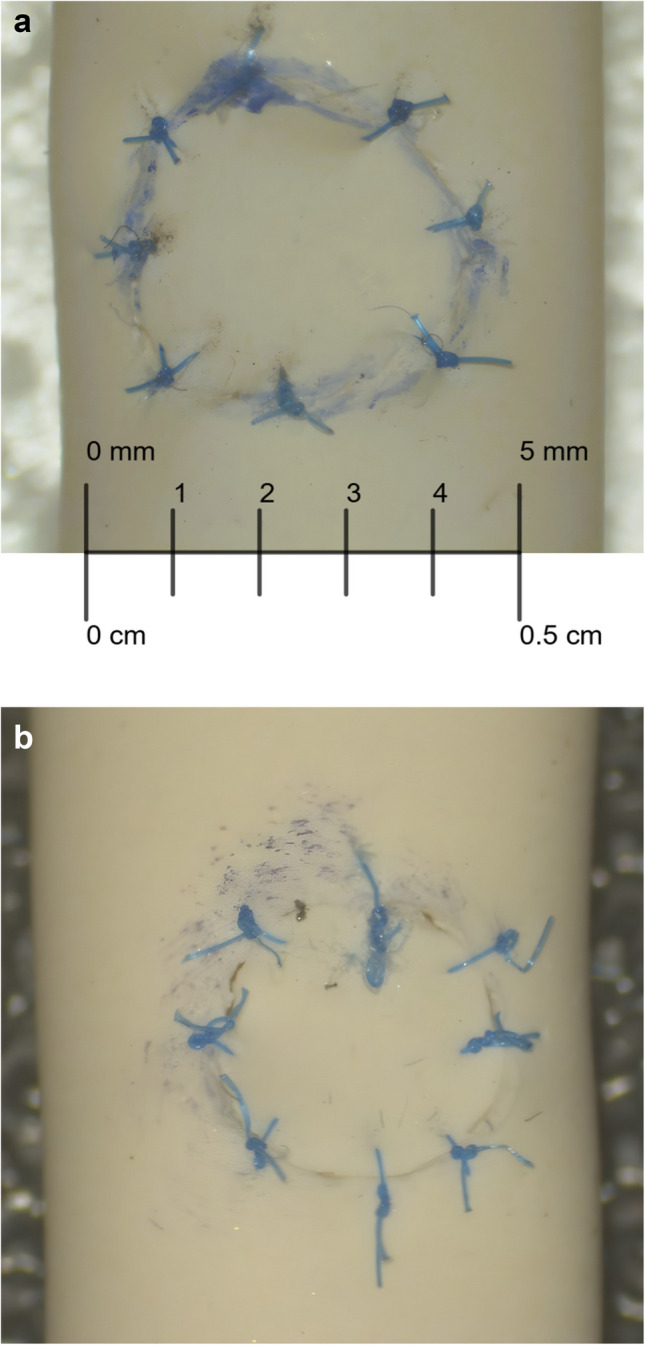
**a** A student’s high-quality last attempt at an 8-stich test (the flap is 4 mm in diameter). Notice that all knots are tight, thread length is consistent, and bite length is mostly consistent, other than in the knot located at 7:30. **b** A student’s first attempt at an 8-stich test (the flap is 4 mm in diameter). Notice the poorly tied knots and the uneven bite length and thread length

A microsuturing test task was designed to measure fine motor skills and handling of microsurgical instruments. The intervention group (*n* = 7) carried out the same test task at the baseline, after 20 h and after 40 h of training. The test task was performed on a latex tube (diameter 5 mm). First, a circular flap (diameter 4 mm) was cut out with microscissors and then re-attached with eight symmetrical 7–0 interrupted sutures (Fig. 1). The needle was handled using a micro needle holder in the dominant hand and jeweler’s forceps in the non-dominant hand.

The suturing tasks were graded based on (i) time and (ii) quality of work by a single researcher (AY). The parameters used for grading suture quality are shown in Table 1. Suturing quality was analyzed as a sum of these parameters and graded between 0–40 points, with 0 being the worst result and 40 the best.

**Table 1:** Variables for grading microsuturing task quality

Length	Is the shorter thread end more than half the length of the longer one? If yes, + 1 point per suture. If no, + 0 points. MAX 8 points
Length Compared to Next	Is the average suture end length in the suture with the shortest ends more than half the suture end length of the next suture? If yes, + 1 point. If no, + 0 points. MAX 8 points
Bite	Is the distance between the disc edge and the entry point of the needle roughly equal to the distance between the disc edge and the exit point of the needle? If yes, + 1 point. If no, + 0 points. MAX 8 points
Space	Comparing the distance (measured along the outer rim of the cut-out disc) between a suture and the ones bordering it, is the shortest distance longer than half the length of the longer distance? If yes, + 1 point. If no, + 0 points. MAX 8 points
Tightness	Can one see through the knot? If yes, + 0 points. If no, + 1 points. MAX 8 points

Suturing quality was analyzed as a sum of these parameters and graded between 0–40 points, with 0 being the worst result and 40 the best

##### Microscraping test task (intervention group)

**Fig. 2:**
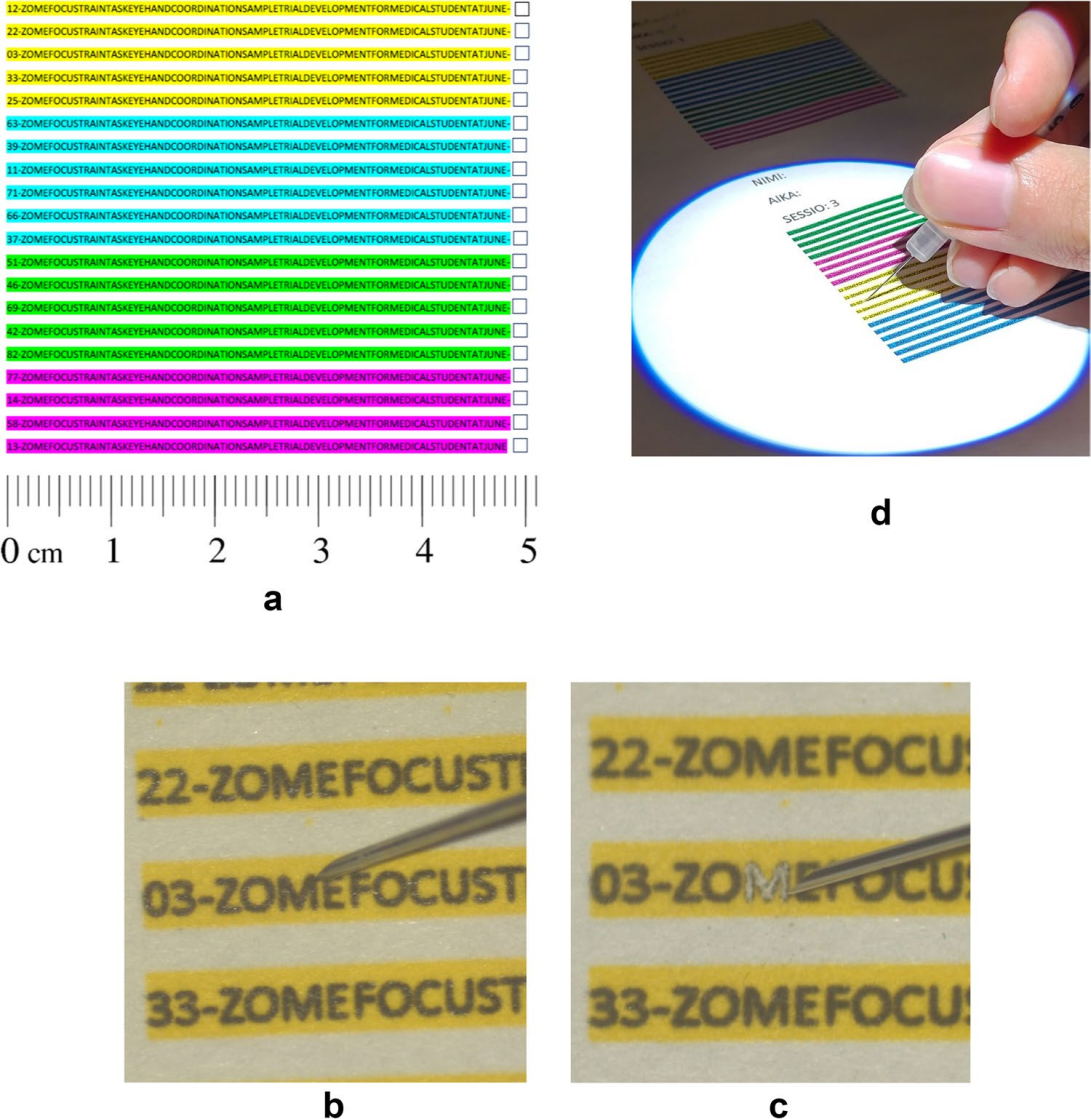
**a** Scraping test layout. Height of individual letters is 0.5 mm. **b** A student starting to scrape the letter M (close-up). **c** The student has scraped the letter (close-up view). **d** The student has scraped the letter (longer-distance view)

A microscraping task was developed to evaluate hand–eye coordination (Fig. 2). The idea of the task was to use a small injection needle to scrape off printed letters from a sheet of paper under a microscope. Each print had 20 rows, with 82 black letters per row on a colored background. The letter height was 0.5 mm (Calibri, 2-point font). Each row started with a different number, e.g. 58. The assignment was to count under the microscope the appropriate letter (e.g. 58th) starting from the left and then to scrape its black pigment off using a small insulin needle without perforating the paper or going outside of the borders of the letter. With one letter scraped off, one would move to the next row until completing all the 20 rows. The intervention group (*n* = 7) completed four of these tasks every week for four weeks (total 16 times). Each participant performed a maximum of one task per day.

All the performances were analyzed by a single researcher (AY). The scraping results were graded based on (i) time and (ii) quality of the work. The parameters used for grading scraping quality are shown in Table 2. Scraping quality was analyzed as a sum of these parameters and graded between 0–45 points, with 0 being the worst result and 45 the best.

**Table 2:** Variables for grading scraping task quality, using five randomly selected rows from each scraping task

Confinement	Has the student restricted their scraping to the letter (black ink), or has the external space been damaged? 3 = excellent confinement, 2 = slight damage to the external space, 0 = significant issues in confining the scraping. The difference in quality between the latter two grades is so significant as to warrant the omission of a grade of 1
Filling	Has the student scraped the entire letter? 3 = yes, 2 = small unscraped areas, 0 = significant unscraped areas. The difference in quality between the latter two grades is so significant as to warrant the omission of a grade of 1
Piercing	Has the student damaged the surface of the paper when scraping it? No = 3, Yes = 0. The grades of 1 and 2 are omitted to render the scale of this variable comparable to the others

Scraping quality was analyzed as a sum of these parameters and graded between 0–45 points, with 0 being the worst result and 45 the best

##### Exoscope test task for both intervention and control groups

The exoscopic test task was carried out by both the intervention (*n* = 6) and the control groups (*n* = 20). One trainee from the intervention group did not participate in the exoscope task. The intervention group was halfway (i.e. 20 h) through their microsurgical training program by the time they did the exoscopic test task. We had designed previously a specific artificial microsurgical model to measure complex depth perception and hand–eye coordination in a microsurgical field [26]. The same model was used in this experiment. The aim of the test task is to pass a microneedle attached to a 6–0 thread as fast as possible through six miniscule discs concealed beneath a styrofoam ring torus using two identical jeweller’s forceps. After a brief, on-site tutorial on exoscope use, and a few minutes of free practice, each participant completed the test task thrice in a row and we measured the time for task completion.

### Data analysis

Due to the limited sample size, we determined statistical significance only for the analysis of variance using SAS statistical software version 8.3. We considered a p value less than 0.05 to indicate statistical significance.

## Results

### Microsuturing and scraping test tasks (intervention group)

#### Microsuturing task

**Fig. 3:**
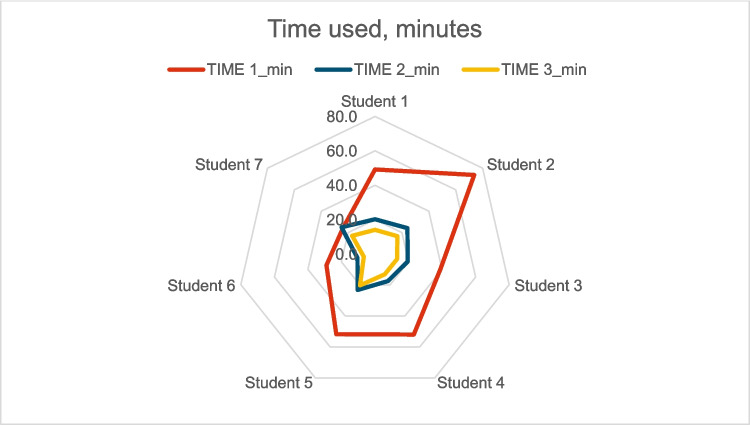
Development in suturing time. The smaller the value, the better the performance. TIME 1 (red) = baseline time, TIME 2 (blue) = time after 20 h training and TIME 3 (yellow) = time after 40 h training

**Fig. 4:**
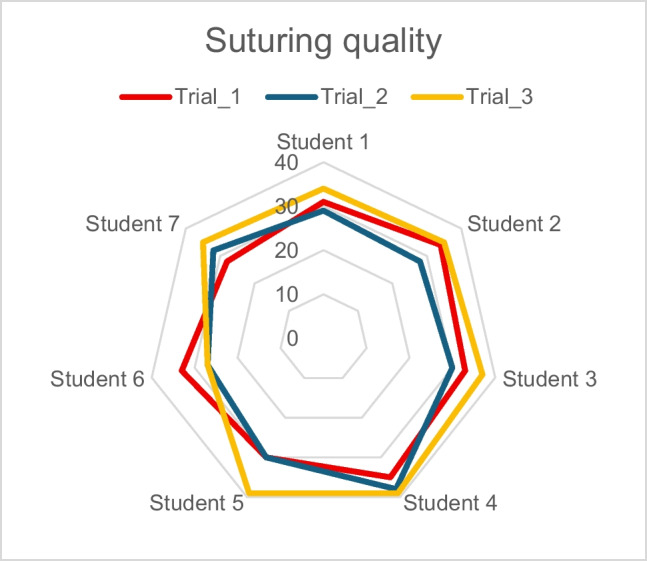
Development in suturing quality. A greater value indicates better performance. Trial 1 (red) = baseline quality, Trial 2 (blue) = quality after 20 h training and Trial 3 (yellow) = quality after 40 h training. Suturing quality is a sum of the Length, LCN (Length Compared to Next), space, tightness and bite parameters. The maximum score for suturing quality is 40 p

Median duration for the microsuturing task improved from 44 min (IQR 22) to 21 min (IQR 6) during the first 20 h of training and further to 14 min (IQR 4) after 40 h (Fig. 3). The quality of sutures did not decline as the trainees got faster (Fig. 4). Suture quality evaluation (Table 1 parameters) resulted in 32/40 points at the baseline (IQR 4) and suture quality remained similar after 20 h (31/40 points, IQR 4) and 40 h of training (35/40 points, IQR 5). Even the trainees with the most improvement in task duration were able to maintain their suture quality. Furthermore, six of seven trainees in the intervention group achieved their personal best quality scores in the final task after 40 h of training, with three scoring near full points (> 35) for quality (39/40, 39/40 and 37/40 respectively).

#### Microscraping task

**Fig. 5:**
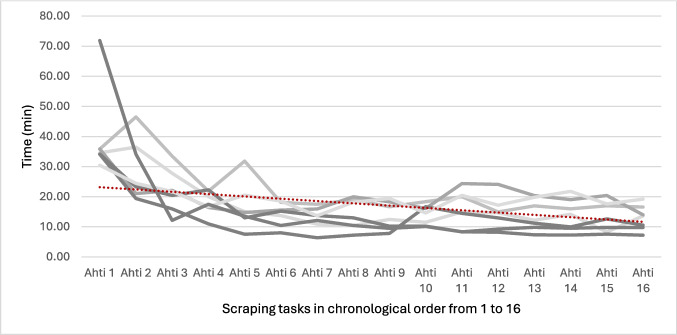
Development of the students in scraping task completion time

**Fig. 6:**
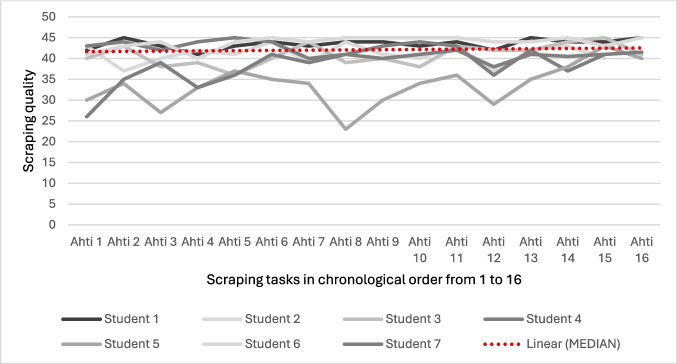
Development of the students in scraping quality as a composite variable of filling, confinement and piercing. The MAX value of the composite variable is 45 p

The median duration of the microscraping task improved from 40 min (IQR 2) to 15 min (IQR 7) during the first 20 h of training and remained similar (13 min, IQR 7) after 40 h (Fig. 5). The quality of work was high and did not decline as the students got faster (Fig. 6). Scraping quality (Table 2 parameters) improved from 38/45 points (IQR 13) to 41/45 points (IQR 5) during the first 20 h of training and then remained the same (42/45 points, IQR 5) after 40 h.

### Exoscope task (intervention and control groups)

**Fig. 7:**
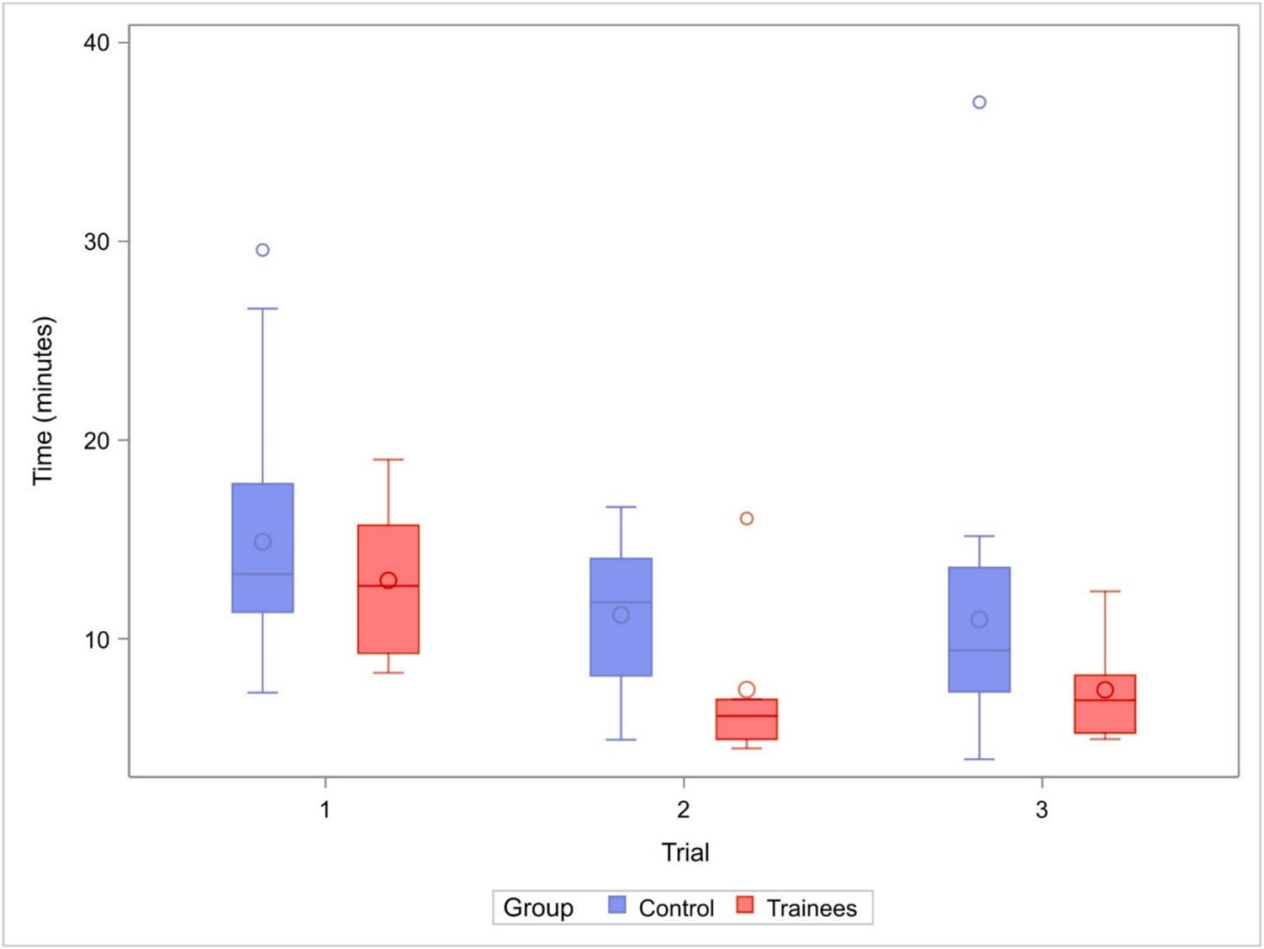
The duration of the exoscope-assisted microsurgical task in the control and intervention group

The intervention group demonstrated better microsurgical skills in the exoscope-assisted microsurgical task than the control group (Fig. 7). The intervention group took a median of 12 min 40 s (IQR 6) to complete the first run, 6 min 7 s (IQR 2) to complete the second run and 6 min 54 s (IQR 3) to complete the third run. Meanwhile, the control group took a median of 13 min 15 s (IQR 6) to complete the first run, 11 min 48 s (IQR 6) to complete the second run and 9 min 24 s (IQR 6) to complete the third run. Taking the length of all runs into account, the mean values differed significantly between the two groups with the intervention group performing better (*p* = 0.04). The 95% confidence interval for the difference in means was −5.73 to −0.47.

## Discussion

Supporting our first hypothesis, the 40-h laboratory training program led to clear and rapid improvement in basic microsurgical skills among microsurgical novices. Most of the progress occurred within the first 20 h, after which the learning curve started to plateau. Performance improved in both microsuturing and microscraping, with participants in the intervention group becoming faster while maintaining consistent quality. While further training may be needed to improve suturing quality, six of the seven trainees in the intervention group achieved their personal best quality scores in the final task after 40 h of training, with three scoring near full points for quality. Furthermore, the skills gained just during the first 20 h of microscope-based training transferred to an unfamiliar exoscope task, suggesting enhanced fine motor control independent of device-specific experience.

In line with our second hypothesis, skill acquisition plateaued toward the end of the course, indicating a potential threshold for early competence. The different training and test tasks varied in their level of complexity. As a result, the plateau in performance was reached earlier in simpler tasks such as scraping, whereas in suturing, which involves more complex movement sequences, skill acquisition continued for longer. In total, our findings suggest a 40-h laboratory training course or even a slightly shorter one may be sufficient to achieve baseline proficiency in basic microsurgical tasks. Longer training may be useful in increasing suturing quality and decreasing suturing time.

The effects of intensive microsurgical training courses on educational models have been previously investigated to some extent and deemed beneficial. Onoda et al. organized microsurgical training for 29 medical students [18]. The training began with suturing of silicon tubes and progressed to anastomosis training in chickens and rats. It took 7–8 h of daily practice over 15 days for students to successfully perform microvascular anastomoses in animal models. Similarly, in a 14-day laboratory course on microvascular techniques (including 100 min daily training), 59 medical students sutured a coronary vessel and prepared a coronary vein under a microscope better than 19 surgeons with varying clinical experience [16]. Both the students and the surgeons had a similar amount of training, and no cardiac surgeons were included. In another study medical students (*n* = 10) achieved equivalent skills to senior surgery residents in a tissue-based coronary surgery simulation model, using 7–0 thread without optical magnification, after four months of training [17]. This does not necessarily translate directly to neurosurgery, since learning to skillfully perform surgical tasks under magnification may take longer. Additionally, while macrosuturing does not require optical magnification and therefore differs fundamentally from microsurgical tasks, training on educational models has been shown to improve macrosuturing skills, which suggests that structured simulation may also confer benefits in microsurgical skill development [3, 9, 10, 19, 25]. Furthermore, regarding all laboratory training, proficiency in isolated tasks does not necessarily translate to competence in performing complex, real-life surgical procedures [12].

As we focused on simpler tasks in our study, the plateau phase was achieved much faster. We found that basic microsurgical manual skills can be taught in low-resource environments without using animals for training purposes. Training on animal models naturally provides benefits regarding tissue handling but is much more resource and time consuming. Simple artificial models, such as ours, enable daily training, e.g., in-between other tasks during a workday. While the training in our current study focused on microsuturing and microscraping, future training programs for neurosurgical trainees could benefit from incorporating a broader range of operative skills, such as drilling and tissue handling [6, 14, 20, 24]. The training provided in the 40 h is not sufficient to allow trainees to master complex operating techniques but merely learn the basics of handling instruments under a microscope.

Skill acquisition may occur more rapidly in novice residents than in medical students, such as those who participated in our study. In a 5-year study, 188 medical students trained by completing 10 stitches multiple times in a line of 50 mm in length on a rubber pad under a microscope [8]. 75 postgraduate physicians, who lacked prior experience in microsurgery, outperformed the students in the course.

We acknowledge some limitations. First, we prioritized high-quality individual training and the overall duration of training over a large sample size. This resulted in a rather small sample size and limited the use of statistical analysis. Second, the test tasks were designed to target basic skills, and they did not convey the complexity of actual clinical scenarios. This resulted in participants having consistently high scores on microsuturing and microscraping quality. However, the simple design of the tasks makes them easily reproducible and suitable for repeated practice and progressive skill refinement. This may help students maintain consistent motivation during training better than complex tasks could. In laparoscopic training, the Fundamentals of Laparoscopic Surgery (FLS) manual skills exam includes simple tasks such as peg transfer and pattern cutting, after which trainees progress to intracorporeal suturing [5, 23, 27]. Similarly, we believe that microsurgical training should follow a stepwise approach: once students achieve efficiency in basic skills, progression to more demanding tasks such as microanastomosis is appropriate. Slightly more complex tasks may also be considered for novices in future studies. Third, the differences in completion time alone may be insufficient to comprehensively grade the students’ performance in microsurgical training. Although completion time was the most important indicator of skill acquisition, our assessment was not based on it alone. Even though the changes in suture quality were scarce, six of the seven trainees in the intervention group achieved their personal best microsuturing quality scores in the final task after 40 h of training. Fourth, baseline data (of the exoscope task) comparing the intervention and control groups prior to training was not collected as the exoscopes were not available at the beginning of the intervention group’s training. Fifth, it would have been optimal to have the intervention group perform the exoscope task also at the end of the 40-h training. This was not possible as the exoscopes were then not available, having been scheduled for surgical procedures. Sixth, only the exoscope task included a separate control group; for the microscraping and suturing tasks, the intervention group’s initial trials served as controls. Seventh, the students in the intervention group may have had high motivation in learning microsurgical techniques, potentially introducing a bias that overestimates the training's effectiveness. However, similar training would optimally be directed to first-year neurosurgery residents, who would likely be similarly motivated. Eighth, one trainee was unable to take part in the exoscope task.

## Conclusions

Novices demonstrated fast progressive improvement in basic microsurgical skills after laboratory training. Even less than 40 h of structured practice may be enough for surgical novices to learn the most basic microsurgical skills prior to assisting in the operating room. We recommend introducing structured microsurgical laboratory training at the beginning of surgical residency, when the skills transfer immediately to clinical practice.

## Data Availability

Our results are found in the manuscript file. However, we are willing to share the full data if contacted. We will share no data allowing identification of the participants.
